# Characterization, Knockdown and Parental Effect of *Hexokinase* Gene of *Cnaphalocrocis medinalis* (Lepidoptera: Pyralidae) Revealed by RNA Interference

**DOI:** 10.3390/genes11111258

**Published:** 2020-10-26

**Authors:** Muhammad Shakeel, Juan Du, Shang-Wei Li, Yuan-Jin Zhou, Naeem Sarwar, Syed Asad Hussain Bukhari

**Affiliations:** 1 Provincial Key Laboratory for Agricultural Pest Management of Mountainous Regions, Institute of Entomology, Guizhou University, Guiyang, Guizhou 550025, China; Shakeelagri1947@gmail.com (M.S.); juandudj@163.com (J.D.); zyj18786607036@163.com (Y.-J.Z.); 2 Department of Agronomy, Bahauddin Zakariya University, Multan 60800, Pakistan; naeemsarwa@bzu.edu.pk (N.S.); asadbukhari@bzu.edu.pk (S.A.H.B.)

**Keywords:** *Cnaphalocrocis medinalis*, hexokinase, RNAi, ds*CmHK*, parental RNAi

## Abstract

Hexokinase (HK) is a key enzyme in chitin biosynthesis in insects and plays an important role in development and energy regulation. It also performs a crucial role in the synthesis of Glucose-6-phosphate and its putative functions are studied via injection of dsRNA corresponding to the *hexokinase* gene from *Cnaphalocrocis medinalis* (*CmHK*). This study was designed to analyze the characteristics and expression patterns of HK-related genes in various tissues of *C. medinalis* at different developmental stages. The *CmHK* ORF is a 1359 bp in length, encoding a protein of 452 amino acids, with homology and cluster analysis showing that *CmHK* shares an 85.11% sequence similarity with hexokinase from *Ostrinia furnacalis.* *CmHK* was highly expressed in the ovary and in the fifth instar larvae. Injection of ds*CmHK* significantly suppressed mRNA expression (73.6%) 120 h post-dsRNA injection as compared to a control group. The results demonstrated an increased incidence of larval and pupal mortality of 80% and 78%, respectively, with significant variation in the sex ratio between males (68.33%) and females (35%), overt larval deformities, and a reduction in average weight gain observed 120 h post-dsRNA injection. In addition, ds*CmHK*-injected *C. medinalis* showed a significant reduction in ovulation per female and larval hatching rate, along with increased larval and pupal mortality and variation in male and female emergence over three generations (G1, G2, and G3). Taken together, the outcomes of the study provide a foundation to study gene function and a new dimension to control *C. medinalis* by transgenic RNAi technology.

## 1. Introduction

The cuticle is an integral part of the exoskeleton that plays a vital role in the growth and development of an organism. In insects, it plays a pivotal role in protecting them against adverse environments, pathogens, parasites, hazardous chemicals, and also provides structural support and movement [1]. Insects periodically shed their old cuticle or cuticular parts and replace them with new ones, especially during molting from one stage to another in which chitin plays a crucial role [1]. Chitin is the second most important and widespread amino polysaccharide in nature after cellulose. It is a linear biopolymer β-(1,4)-linked N-acetylglucosamine that is assembled into microfibrils of different length and diameter and is mostly synthesized by nematodes, fungi, protozoan, mollusks, and arthropods [2,3]. In insects, chitin synthesis plays a crucial role in growth and metamorphosis, and also serves as a central component of the embryonic cuticle, trachea and peritrophic membrane (PM), and the extracellular linings of the body [4,5]. Several genes associated with chitin have been reported in ovaries, eggs, and eggshells of various insect species [6]. Therefore, disruption of natural processes of chitin synthesis and degradation can cause abnormalities, leading to death in severe cases [7]. 

Hexokinase (HK) is the first enzyme in the glucose metabolic pathway [8,9]. HK produces multi-functional proteins involved in apoptosis [10] and transcriptional regulations [9]. In the glycolysis pathway, HK serves as an important rate-limiting enzyme that converts glucose to glucose-6-phosphate [11]. In the glycolysis pathway, glucose uses HK as a substrate to convert glucose 6-phosphate to pyruvate in organisms [11]. HK acts as a neurotrophic factor in cytokine neurotransmission [12,13,14]. HK is also the second most important enzyme in the chitin biosynthesis pathway [15]. HK was first studied in *Nematocida parisii* [16] and has also been reported in *Paranosema locustaes* [17,18,19]. HK was also present in the host’s indirect immune-fluorescence assay (IFA) [17,18]. The primary function of HK has been observed in *Trachipleistophora hominis*, where it serves as a regulator to enhance ATP synthesis on the surface of parasites [20]. 

RNA interference (RNAi), has been reported as an effective gene silencing technique in eukaryotic organisms [21]. It is an endogenous post-transcriptional gene silencing (PTGS) mechanism used to regulate gene expression at the mRNA level with a highly conserved mode of action [22,23]. Double-stranded RNA (dsRNA) is turned into siRNA (small interfering RNA) which causes rapid degradation of mRNA [24]. Specific degradation of mRNA occurs in the cytoplasm resulting in the silencing of normal gene function. Firstly, RNAi was found in *Caenorhabditis elegans* [25]. It has been reported in fungi, plants, and animals including insects [26,27,28,29,30]. In insects, it was studied in *Plutella xylostella*, *Spodoptera exigua,* and *Manduca sexta* [31], *Tribolium castaeum*, *Gryllus bimaculatusa* along with their progenies [32,33], and *Henosepilachna vigintioctopunctata* [34]. RNAi has two types that are systemic RNAi and environmental RNAi in which siRNA is introduced by injection method, and by oral administration in the entomological research. However, dsRNA application mostly affects the efficiency of RNAi. Direct injection of dsRNA to insect hemocoel causes gene silencing and is considered a successful method for controlling the desired gene function [27]. Higher larval mortality was observed in *S*. *exigua* after microinjection of two highly preserved genes, *hexamerin1* and *protein1,* as compared to the controlled conditions [35]. At present, the microinjection method proves relatively effective for the management of *Spodoptera litura* [36], *M*. *sexta* [37], and *Bombyx mori* [38]. In the case of *Helicoverpa armigera*, delayed pupal developments were observed after the injection of HK inhibitor Deoxy-2-glucose (DOG) [39]. It appears that HK dysfunction inhibits the chitin formation that may lead to insect abnormality, stunted growth, and delayed pupation. Up to now, functions of HK have not been investigated in *Cnaphalocrocis medinalis*. Additionally, *C. medinalis de novo* assembly transcriptome data have also been published [40]. Therefore, RNAi strategy could be a useful tool to control *C. medinalis* and its gene functions.

RNAi is mainly categorized into parental RNAi, embryonic RNAi, and larval RNAi. However, parental RNAi (pRNAi) is mostly used to study gene expression [41,42,43,44]. pRNAi was first achieved by injecting dsRNA into the insect body by the gene silencing of female offspring and also tested against various genes of insect embryos including the *distel-less* gene for the formation of limbs in *Nilaparvata lugens* and *T. castaneum* [25,32,45], the *maxillopedia* gene for maxillary and labial palps formation in *T. castaneum* [32,46], the transformation genes for sex differentiation in *G. bimaculatus* and *T. castaneum* [41,47,48], and the *hunchback* gene for the formation of axial patterning in *Diabrotica virgifera virgifera, Oncopeltus fasciatus*, and *Acyrthosiphon pisum* [49,50,51,52]. However, the effects of pRNAi on the *HK* gene have not been reported in *C. medinalis*.

The rice leaf folder (*Cnaphalocrocis medinalis*) (Lepidoptera: Pyralidae), is an adaptive and economically important rice pest in most Asian countries including China [53]. *C. medinalis* possesses complete metamorphosis and undergoes four developmental stages, that is, egg, larva, pupa, and adult. Eggs are oval in shape and creamy white, laid in batches along the midrib of the leaf blade with 0.90 mm length and 0.39 mm width. Larvae have five stadiums [54]. Newly emerged larvae are light- or greenish-yellow with 1.5–2 mm length and 0.3 mm width, while the fifth instar larvae are 20–25 mm long and greenish-yellow in color [54]. *C*. *medinalis* larvae attack all stages of the rice plants by feeding on the rolled leaves. They scratch chlorophyll, inhibit photosynthesis, and reduce grain yield. During an epidemic situation, *C. medinalis* larvae cause a 30 to 80% decline in rice yield [55,56]. At present, the control of *C. medinalis* is achieved through extensive use of various chemical insecticides [57]. The cultivation of rice at a large scale, application of various insecticides, and fertilizers seem to favor *C. medinalis* population outbreaks [58,59,60]. Zhang et al. revealed that *C. medinalis* has evolved a high level of resistance to metaflumizone, tebufenozide, chlorantraniliprole, chlorpyrifos, indoxacarb, tebufenozide, and monosultap [61]. Previously, it has been reported that behavioral and physiological modifications can enhance detoxification and reduce target sensitivity in *C. medinalis* [62]. Therefore, it is an urgent need to identify environmentally safe methods to control *C. medinalis*. Chitin synthetic pathway is found in insects but is not present in vertebrates [63]. Therefore, we have considered chitin synthesis genes as target sites to control *C. medinalis*. 

In the present study, the *hexokinase* gene from *C. medinalis* (*CmHK*) (Accession Number: MN612078) was identified from the transcriptome database, and its spatial and temporal expressions were analyzed. Expression patterns of *CmHK* can be suppressed by injecting dsRNA in *C. medinalis*. Furthermore, the effects of pRNAi have been observed in three generations (G1, G2, and G3) of the injected *C. medinalis*. However, the results of the current study indicate that the effects of dsRNA injection targeting the *CmHK* gene was induced a significant phenotypic disruption, larval and pupal mortality, disproportionate in male to female sex ratio, and their effects also observed in three generations (G1, G2 and G3) of *C. medinalis*. 

## 2. Materials and Methods

### 2.1. Insect Rearing 

*C. medinalis* adults were collected from paddy fields of Guizhou Province, China in 2019, and maintained at the Institute of Entomology, Guizhou University. Newly emerged larvae were derived from the progeny of one pair of mated insects, which were reared on rice seedlings in a chamber at 26 ± 1 °C, 75 ± 5% (RH), and 14:10 h light: dark photoperiod. Four life stages, that is, egg, the first to the fifth instar larvae, pupa, and adults were used in different experiments. 

### 2.2. RNA Isolation, cDNA Synthesis and RT-PCR

The head, midgut, malpighian tubules, fat body, testes, muscle, cuticle, and ovary were dissected from both male and female adults in cold 0.01 M phosphate-buffered saline solution. All samples were stored at −80 °C until required. Total RNA from the whole insect body was isolated using the HP Total RNA Kit (Omega Bio-Tek, Norcross, GA, USA) in accordance with the manufacturer’s protocol. Total RNA was quantified, and purity was assessed using a NanoDrop 2000 spectrophotometer (Thermo Fisher, Waltham, MA, USA). The first-stranded cDNA was synthesized using RevertAid First Strand cDNA Synthesis Kit (Thermo Fisher, Waltam, MA, USA), following the manufacturer’s instructions. The cDNA was then stored at −20 °C until required. The specific primers (Table 1) for reverse transcription-PCR (RT-PCR) were designed based on the fragment from *C. medinalis* transcriptome [40]. RT-PCR was conducted in a 20 μL reaction system including 1 μL of cDNA template, 1 μL of each primer, 10 μL of 2 × Master Mix (Tsingke, Bejing, China), and 7 μL of ddH_2_O. The PCR reaction conditions were as follows: initial denaturation at 94 °C for 1 min, followed by 30 cycles at 94 °C for 30 s, 55 °C for 30 s, and 72 °C for 90 s, and a final extension of 10 min at 72 °C. The expected size of the PCR fragment was purified with a MiniBEST Agarose Gel DNA Extraction Kit (Takara Bio, Beijing, China), and was sequenced by Sangon Biotech (Shanghai, China).

### 2.3. Sequence Retrieval and Analysis

To obtain the *CmHK* cDNA, the sequence of HK from *O. furnacalis* (OfHK: LOC_114357200) was used to search against the transcriptome database of *C. medinalis* with tblastn. We identified one *C. medinalis* cDNA unigene (CL823) presenting significant similarity to OfHK (85.11%). The *CmHK* cDNA was further verified by RT-PCR and by Blastx search against the NCBI (National Center for Biotechnology Information) GenBank based on insect HKs. The sequence was analyzed using the ORF finder at the NCBI (https://www.ncbi.nlm.nih.gov/orffinder). Molecular weight and isoelectric point (pI) of CmHK were analyzed using ProtParam (http://web.expasy.org/protparam), and the single peptide was predicted using SignalP 5.0 (https://services.healthtech.dtu.dk/services.php?/SignalP-5.0). 

The transmembrane helices in CmHK were analyzed at TMHMM server v.2.0 (https://services.healthtech.dtu.dk/services.php?/TMHMM-2.0), and phosphorylation sites were predicted at KinasePhos (http://kinasephos.mbc.nctu.edu.tw). Multiple sequence alignment of CmHK with other insect HKs was performed using Clustal Omega (https://www.ebi.ac.uk/Tools/msa/clustalo). Glycosylation sites were estimated by using NetOGlyc 4.0 (https://services.healthtech.dtu.dk/service.php?NetOGlyc-4.0) and NetNGlyc 1.0 (https://services.healthtech.dtu.dk/service.php?NetNGlyc-1.0). A three-dimensional (3D) structural homology modeling of CmHK was analyzed by using SWISS-MODEL (https://swissmodel.expasy.org) and then visualized with PyMOL 2.3.4 (Schrodinger, New York, NY, USA). 

### 2.4. Phylogenetic Analysis of CmHK

The phylogenetic tree was constructed using MEGA X with the neighbor-joining method. Bootstrap analysis was carried out (1000 replicates) to calculate the percentage of the replicate tree. The insects that hexokinases were from included: *C. medinalis* (Cm), *Delia antiqua* (Da), *Drosophila navojoa* (Dna), *Drosophila novamexicana* (Dno), *Drosophila busckii* (Db), *Frankliniella occidentalis* (Fo), *Anopheles sinensis* (As), *Anopheles darling* (Ad), *Aedes aegypti* (Aa), *Cryptotermes secundus* (Cs), *Zootermopsis nevadensis* (Zn), *Culex quinquefasciatus* (Cq), *Antheraea pernyi* (Ap), *Galleria mellonella* (Gm), *Hyposmocoma kahamanoa* (Hk), *Amyelois transitella* (At), *B. mori* (Bm), *Bombyx mandarina* (Bma), *M. sexta* (Ms*), Papilio machaon* (Pm), *Papilio polytes* (Pp), *Papilio xuthus* (Px), *O. furnacalis* (Of), *Pieris rapae* (Pr), *Bicyclus anynana* (Ba), *Vanessa tameamea* (Vt), *Trichoplusia ni* (Tn), *H. armigera* (Ha), and *S. litura* (Sl).

### 2.5. Tissue and Developmental Expression Patterns of CmHK

The expression of *CmHK* was assessed in all dissected tissues of *C. medinalis.* The expression of *CmHK* was quantified at different stages, that is, eggs, five larval instars, pupae, and male and female adults, collected from insects reared in the laboratory. Total RNA was extracted from both whole-body samples and tissues by using the Total RNA Kit (Omega Bio-Tek, Norcross, GA, USA, following the instructions provided by the manufacturer. Using RevertAid First Strand cDNA Synthesis Kit (Thermo Fisher, Waltam, MA, USA), the first-stranded cDNA was synthesized from tissues and all developmental stages, respectively. *CmHK* gene-specific primers were designed using Primer Premier 6.0 (Premier Biosoft, San Francisco, CA, USA). Real-time quantitative PCR (RT-qPCR) was performed to measure the expression levels of *CmHK* in various tissues and different developmental stages of *C. medinalis*. The reaction mixture included 10 μL of 2x iTaq Universal SYBR Green Supermix (Bio-Rad, Hercules, CA, USA), 1 μL of cDNA template, 1 μL each of the HK-qF and HK-qR primers (Table 1), and 7 μL ddH_2_O in a 20-μL total volume. The amplifications were carried out with the following cycling conditions: one cycle at 95 °C for 2 min, followed by 40 cycles of denaturation at 95 °C for 20 s, 55 °C for 20 s, and 72 °C for 30 s. The *β-actin* gene was used as the internal control. Three biological replicates were performed for both tissues and developmental stages of *C. medinalis*. The relative expression level of *CmHK* at different stages and in different tissues was analyzed by using 2^−∆∆Ct^ method [64]. The significance of differences was determined by the LSD test.

### 2.6. Synthesis and Effect of dsCmHK

The target sequence specific to *CmHK* mRNA was searched by using online RNAi design tools, that is, siDirect (http://sidirect2.rnai.jp) and siRNA at Whitehead (http://sirna.wi.mit.edu). Then, the designed target sequence was amplified using the HK-iF and HK-iR primers with *CmHK* cDNA as the template. The purified PCR product was inserted into pMD20-T vector (Takara Bio, Beijing, China) and then transferred into *E. coli* TOP10 competent cells for sequencing. The clone containing the correct sequence was cultured to extract plasmids for amplifying the target fragments with both the HK-dsF and HK-dsR primers. The PCR product was purified to produce highly concentrated DNA that was used as the template to synthesize the *CmHK* dsRNA using a TranscriptAid T7 High Yield Transcription Kit (Thermo Fisher, Waltham, MA, USA), in accordance with the manufacturer’s protocol. Purification of dsRNA was done using GeneJET RNA Purification Kit (Thermo Fisher, Waltham, MA, USA), according to the manufacturer’s protocol. The integrity of dsRNA was determined on 1% (*w/v*) agarose gel by electrophoresis. The concentration was quantified using a NanoDrop 2000 spectrophotometer (Thermo Fisher, Waltham, MA, USA). The *GFP* dsRNA (ds*GFP*) was used as a control. 

The microinjection of dsRNA was used as a delivery method for the RNAi assay. To silence the *CmHK* gene, a microinjection method was performed using the NT-88-V3 micromanipulator (Nikon, Tokyo, Japan). For injection, the eighth abdominal segment of the fourth instar larvae along with blood flow direction was selected. Then, 0.5 μL (2 μg/μL) ds*CmHK* and ds*GFP* were prepared and injected into the fourth instar larvae. A total of 20 larvae were placed in each experiment with three replicates to check mortality and abnormality, average weight loss, and to measure the mRNA level of expression by RT-qPCR at 24-h intervals. All treated samples were placed on newly emerged rice leaves in a growth chamber under controlled conditions described above. To measure the mortality, larvae that were alive were counted on a daily basis for five days. Furthermore, adults emerging from the treated group were used for parental RNAi experiments.

### 2.7. Parental RNAi

To measure the effect of ds*CmHK* at the progeny level, one newly emerged adult male and one female from treated groups were paired and placed in a transparent plastic box (5.1 cm long, 3.8 cm wide, and 2.9 cm high) with vented lids. A diluted honey solution, soaked in a cotton plug, was placed in the plastic box for food. Plastic boxes were kept in a controlled chamber at 26 ± 1 °C, 75 ± 5% (RH), and 14:10 h light: dark photoperiod. Each pair of insects was allowed to mate for four to five days for oviposition. After egg laying, paired adults were removed from boxes and kept at −80 °C post liquid nitrogen quick freezing to analyze the mRNA level of expression by RT-qPCR. Percentage of eggs laying and eggs hatched per female was counted using ImageJ software [65], and surviving larvae, number of pupae, and male and female emergence were calculated from groups treated with ds*CmHK* and ds*GFP*. These experiments were carried out at three generations (G1, G2, and G3) to verify the pRNAi effects.

### 2.8. Statistical Analyses

The 2^−ΔΔCt^ method was used to analyze the mRA expression levels of *dsCmHK* and ds*GFP* in different tissues and at different growth stages after injection in three progenies (G1, G2, and G3) [65]. The LSD test was used to measure the significance of differences in larval abnormality, larval mortality, weight loss, eggs hatched, pupal mortality, and male and female emergence using SPSS 22.0 (SPSS Inc., Chicago, IL, USA).

## 3. Results

### 3.1. Sequence and Expression Pattern Analyes of CmHK

**Figure 1:**
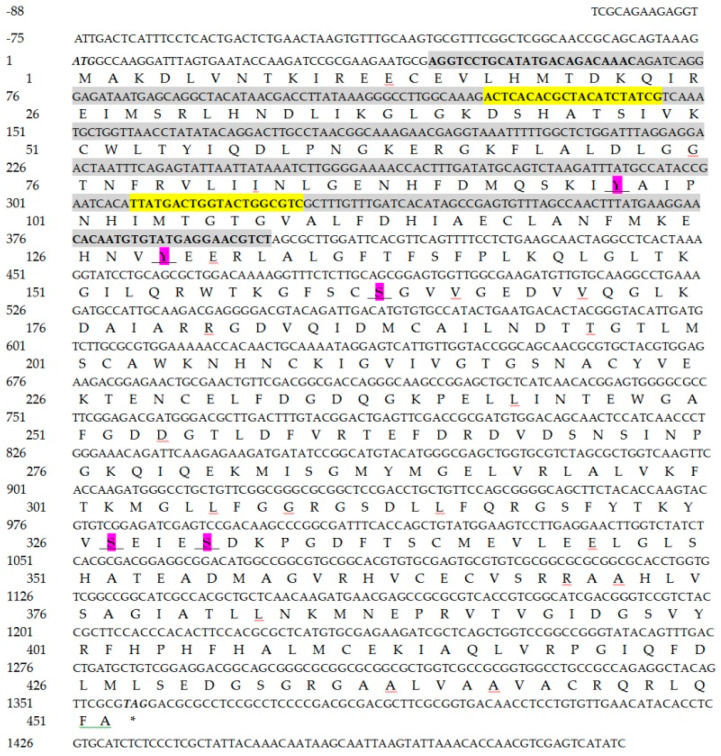
Nucleotide and deduced amino acid sequences of *CmHK*. Start and stop codons are indicated by bold typeface and italic. One conserved domain of *CmHK* used for dsRNA synthesis (44–398 bp) is indicated as grey. Phosphorylated sites are underlined in bold and pink. Primers of ds*CmHK* synthesis are shaded in bold, while RT–qPCR primers are indicated by bold and yellow shading.

**Figure 2:**
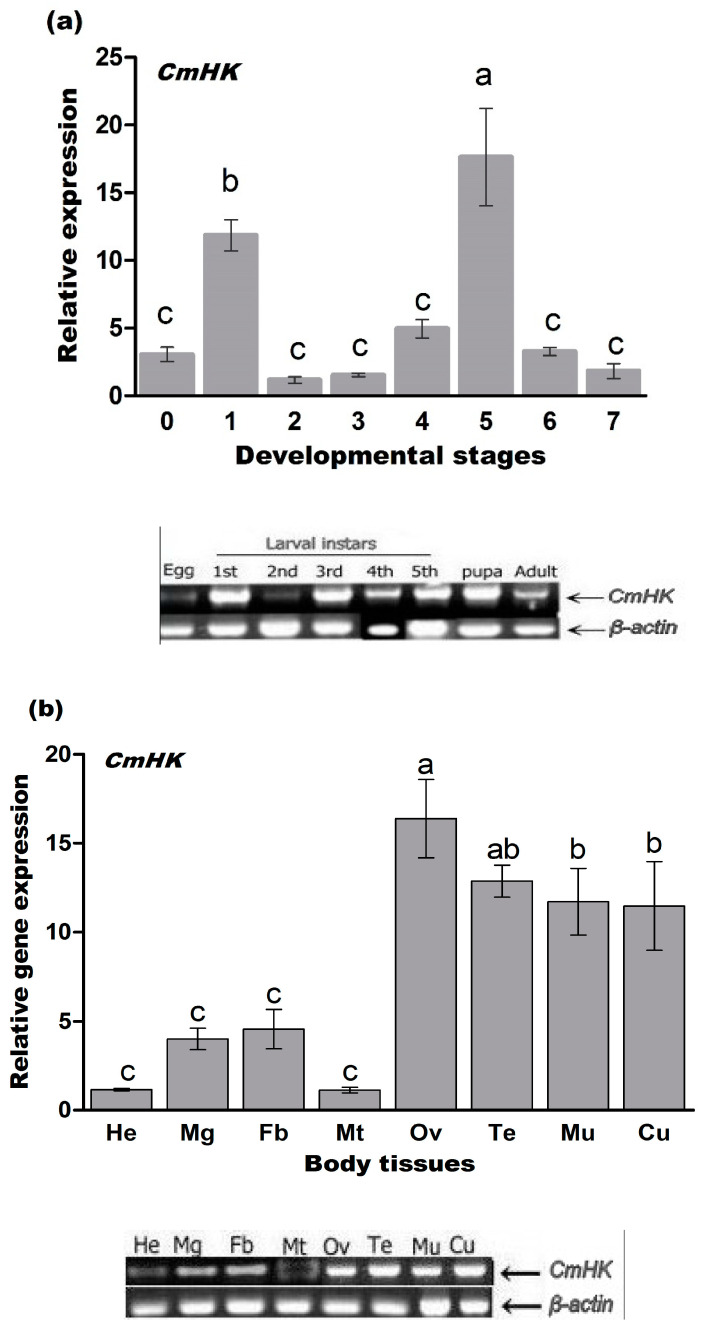
Expression pattern of *CmHK* in different tissues and at different developmental stages of *C. medinalis* adults. (**a**) Expression pattern of *CmHK* in eggs (0), the first to fifth instar larvae (1–5), pupae (6), and adults (7) of C. *medinalis*. (**b**) Expression pattern of *CmHK* in the head (He), midgut (Mg), fat body (Fb), malpighian tubules (Mt), testes (Te), muscle (Mu), cuticle (Cu), and ovary (Ov). Relative mRNA levels of *CmHK* were analyzed using RT-qPCR. *β-actin* is used as an internal control. Each bar indicates the mean ± *SD*, and different letters above each bar represent a significant difference (*p < 0.05*, LSD and ANOVA) from three independent experiments.

The length of the *CmHK* cDNA is 1581 bp, containing an ORF of 1359 nucleotides (nt) that encodes 452 amino acids (aas) (GenBank accession number: MN612078). It contains a 5′ untranslated region (UTR) of 88 bp and a 3′ UTR of 134 bp. The nucleotide and predicted amino acid sequences of *CmHK* are shown in Figure 1. The theoretical isoelectric point of CmHK protein is 5.99 with a molecular weight of 50.06 kDa. CmHK possesses no glycosylation sites and signal peptides or transmembrane structures, and has 5 phosphorylated sites (Figure 1). Additionally, upon the NCBI Blast, *CmHK* showed the highest similarity with *OfHK* of *O. furnacalis* (XM_028310711, 88.37% identity), followed by *MsHK* of *M. sexta* (XM_030167810, 84.75% identity) and *PpHK* of *P. polytes* (XM_013282688, 84.30% identity). This research shows that the fifth instar larvae were defined to have the highest expression level of *CmHK*, followed by the first and fourth instar larvae at different developmental stages (Figure 2a). The expression of *CmHK* was observed in the all tissues tested, with the highest expression level in the ovary (Figure 2b).

### 3.2. Phylogenetic Analysis and 3D Structure of CmHK

**Figure 3:**
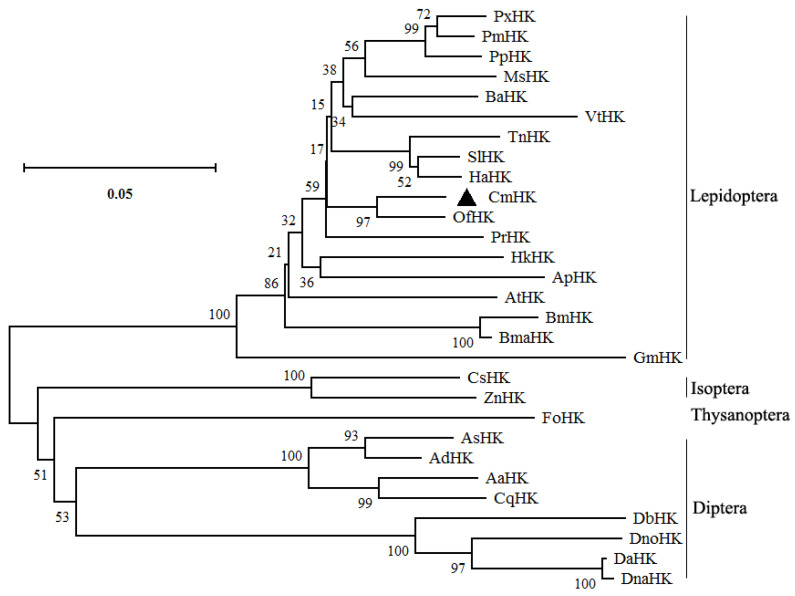
Phylogenetic tree shows the relationship of *CmHK* with other insect hexokinases. These amino acid sequences were analyzed by Clustal Omega. The tree was constructed using MEGA X. The bootstrap test with 1000 replicates shows the percentage of replicate trees in which sequences are clustered. GenBank accession numbers of different insect HKs are as follows: CmHK (MN612078), DaHK (XP_017870606), DnaHK (XP_030244412), DnoHK (XP_030568037), DbHK (XP_017853033), FoHK (XP_026290299), AsHK (KFB44789), AdHK (ETN63660), AaHK (XP_011493158), CsHK (XP_023727905), ZnHK (XP_021941686), CqHK (XP_001850122), ApHK (ATA67117), GmHK (XP_026748941), HkHK (XP_026319325), AtHK (XP_013192105), BmHK (XP_004932650), BmaHK (XP_028033573), MsHK (XP_030023668), PmHK (XP_014362584), PpHK (XP_013138142), PxHK (XP_013173406), OfHK (XP_028166512), PrHK (XP_022115019), BaHK (XP_023938410), VtHK (XP_026496396), TnHK (XP_026733584), HaHK (XP_021197237), and SlHK (XP_022834977).

**Figure 4:**
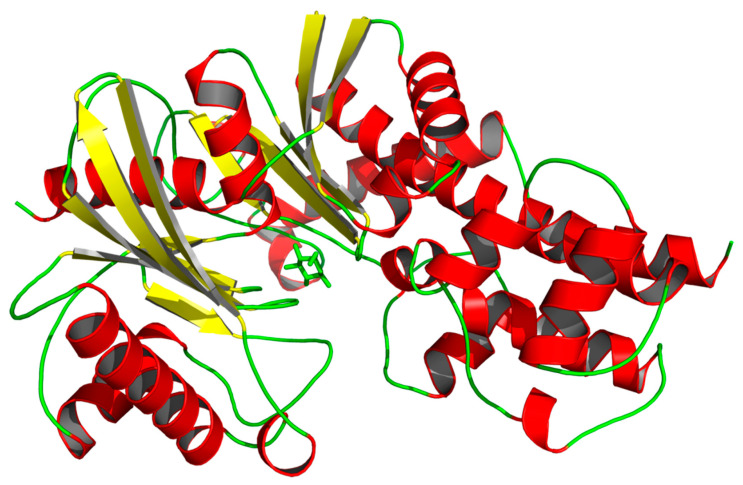
Three-dimensional structure of CmHK. Predictions for CmHK from C. *medinalis* were generated by SWISS-MODEL and visualized with PyMOL 2.3.4 [66]. Red represents α-helices, yellow indicates β-pleated sheets, and green denotes random coils.

A phylogenetic tree was generated based on an aligned amino sequence of various insect hexokinases using MEGA X (Figure 3). Hexokinases from different insect species in different orders were placed in different clusters. CmHK was grouped with OfHK from *O. furnacalis*, suggesting that *C. medinalis* is the closest relative to *O. furnacalis*. Homology modeling revealed that CmHK formed 18 α-helices, 12 β-pleated sheets, and 29 random coils (Figure 4).

### 3.3. Effects of RNAi 

#### 3.3.1. Effects of RNAi on *CmHK* Gene Expression 

**Figure 5:**
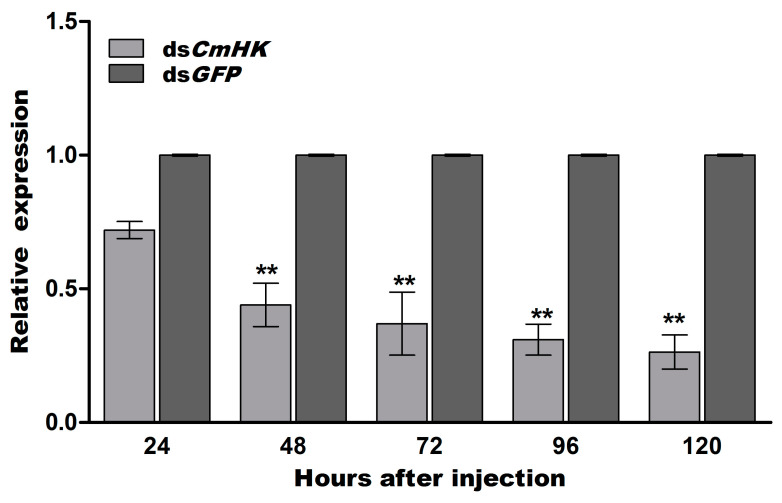
The expression level of *CmHK* after dsRNA treatment at 24-h intervals. Data were shown as the mean ± *SD*. Significant differences are indicated by ** (*p* < 0.05).

To obtain the silencing of the *CmHK* gene achieved by injection of dsRNA in C. *medinalis*, 0.5 μL (2 μg/μL) of ds*CmHK* was tested. Detection of *CmHK* silencing was observed according to different time durations (24 h, 48 h, 72 h, 96 h, and 120 h). A significant silencing of *CmHK* was achieved at 48, 72, 96, and 120 h post-injection duration (*p* < 0.05) (Figure 5).

#### 3.3.2. Phenotypic Effects of RNAi on *C. medinalis*

**Figure 6:**
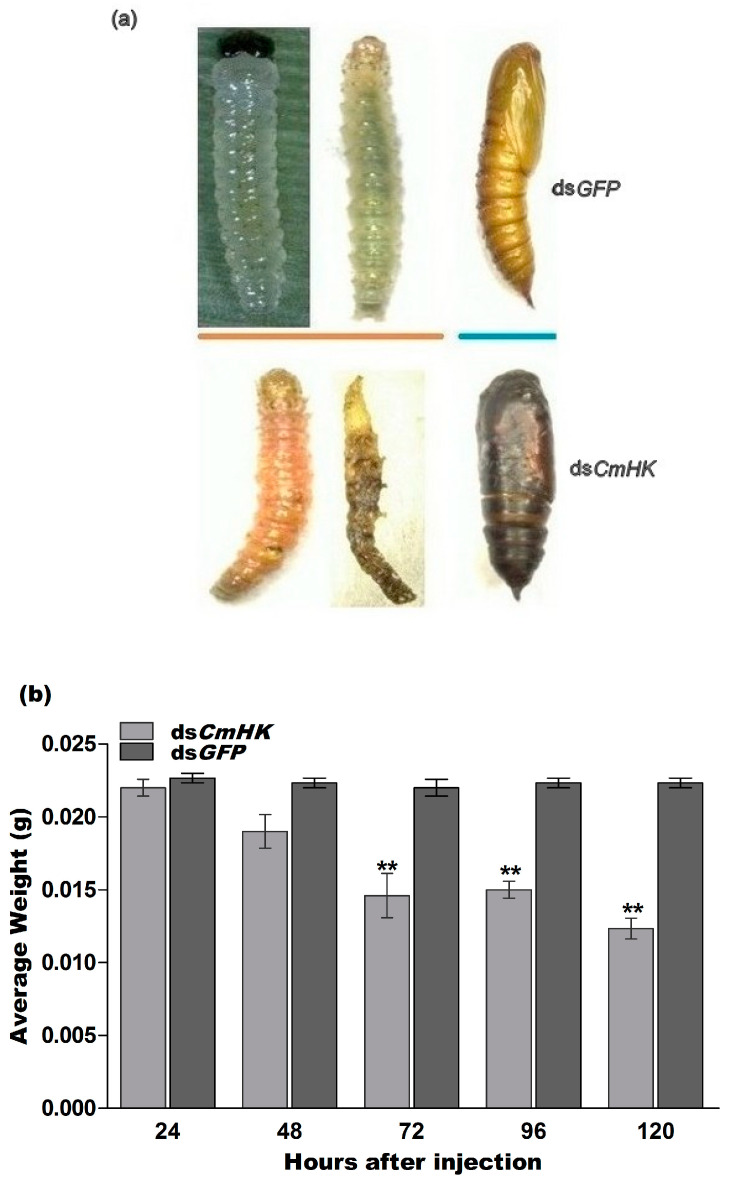

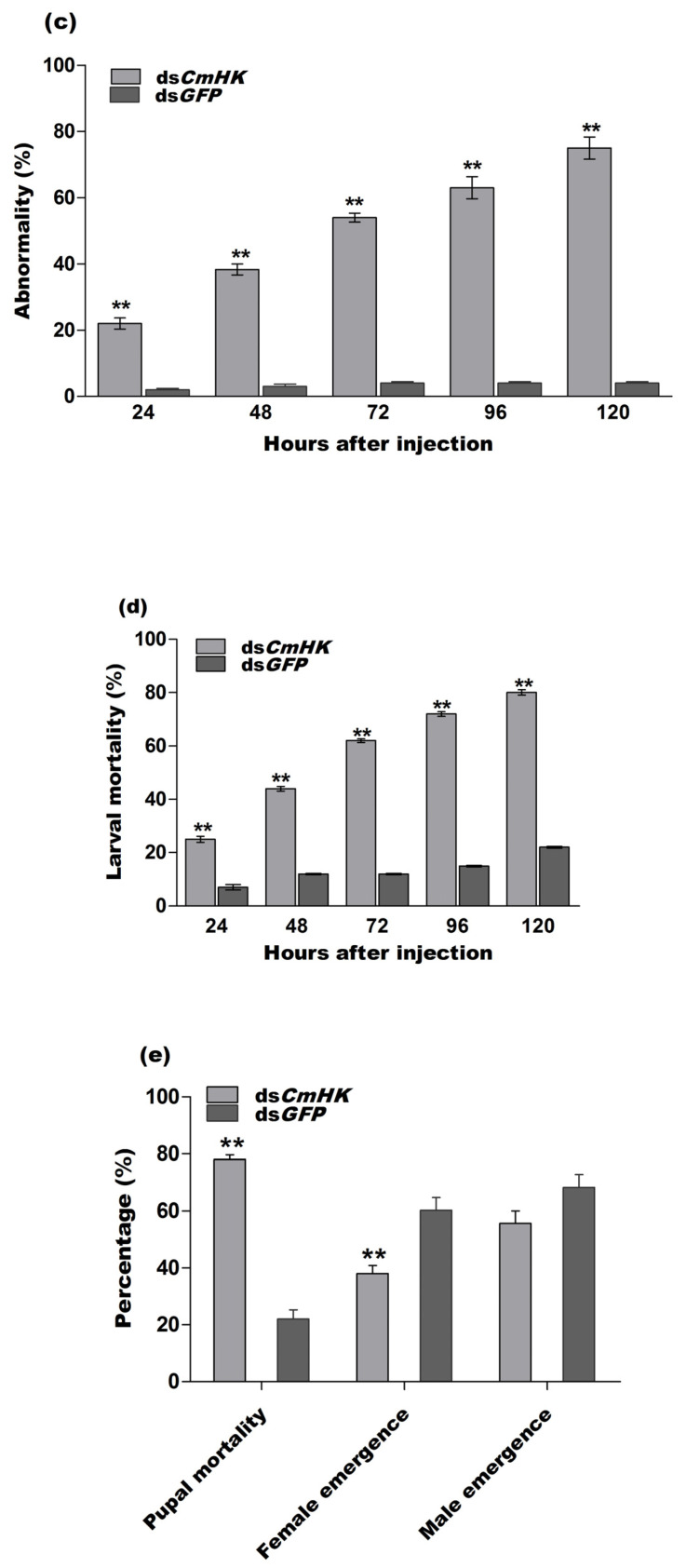
Phenotypic expressions, average weight loss, abnormality, larval and pupal mortality rates, and male and female emergence rates in response to injection with ds*CmHK* as compared to ds*GFP* in *C. medinalis*. Twenty larvae were selected in one group for injection as a replicate. (**a**) Abnormal phenotypic expressions (larvae and pupae). (**b**) Average weight loss at 24-h interval (24–120 h). (**c**) Abnormality rates. (**d**) Mortality rates 24–120 h after ds*CmHK* and ds*GFP* injection. (**e**) Pupal mortality of injected larvae after pupation formation, and male and female emergence ratio. Each bar indicates the mean ± *SD* and significant differences are indicated by ** (*p* < 0.05).

Phenotypic deformities were observed in *C. medinalis* when the *CmHK* gene was silenced by RNAi (Figure 6a). The results also indicated that some larvae did not undergo metamorphosis and hence did not complete the molting process. No phenotypical deformities were observed in the ds*GFP* control. In addition, weight decreased significantly at 72 h, 96 h, and 120 h post-injection duration (Figure 6b). The significant mortality and abnormality rates were 80% and 75%, respectively, at 120 h post-injection with ds*CmHK* (Figure 6c,d). Pupal mortality was 78% in the ds*CmHK*-injected group, and 22% in the ds*GFP*-injected group (Figure 6e). Our results indicated that significant (*p* < 0.05) pupal reduction occurred in the case of the ds*CmHK*-injected insects as compared to the ds*GFP*-injected group. We also studied the variation in sex ratio in the ds*CmHK-* and ds*GFP*-treated groups (Figure 6e). Compared with the control treatment, ds*CmHK* exhibited a significantly higher emergence of male adults than that of female ones at *p* < 0.05. 

#### 3.3.3. Parental RNAi

##### G1-Generation

**Figure 7:**
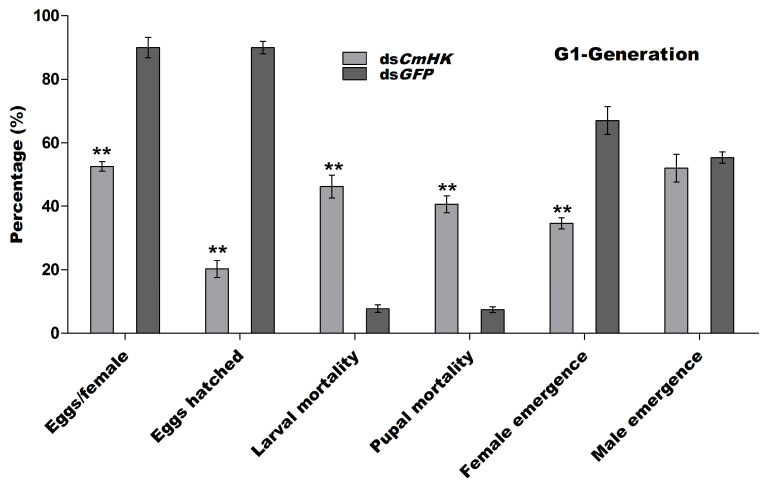
Percentage of eggs per female, eggs hatched, larval and pupal mortality, and male and female emergence rate in G1-generation of *C. medinalis*. Five pairs of male and female adults were selected for the G1-generation experiment. Each bar indicates the mean ± *SD* from the *CmHK* and *GFP* groups. Significant differences are indicated by ** (*p* < 0.05).

Previous research exploring the transmission of the effects of RNAi revealed that in some cases gene knockout was transmitted from treated parents to their progeny [67]. To confirm this phenomenon, five emerged male and female pairs were selected from the groups injected with ds*CmHK* and ds*GFP*, and each pair was kept in a separate transparent plastic box to mate. The total number of eggs was counted per female. Eggs laying and eggs hatched were significantly declined in the G1 generational female as compared to the control. Larval and pupal mortality was significantly higher in the G1 generation as compared to the control treatment. The results showed that hatched larvae and larval mortality were 19% and 41% in G1 ds*CmHK*-treated insects, respectively. While in the G1 control treatment, hatched larvae and larval mortality were calculated as 87% and 7%, respectively. Interestingly, the emergence of males was higher (51.6%) in G1 insects treated with ds*CmHK* than the untreated ones (49.4%) (Figure 7). 

##### G2-Generation

**Figure 8:**
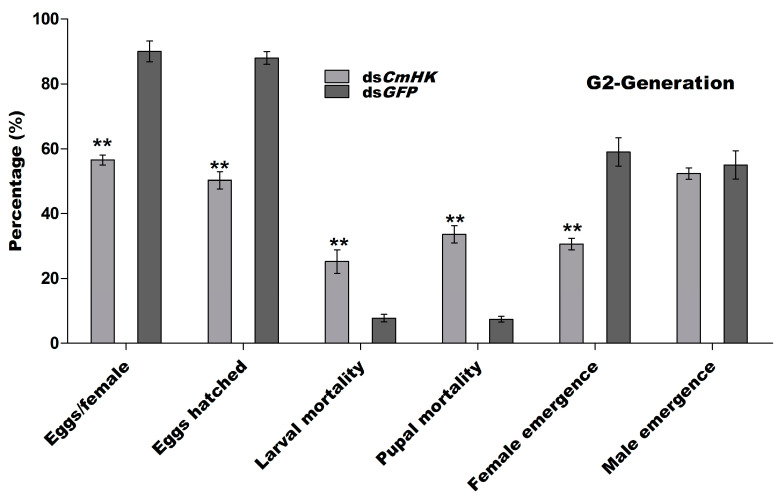
Five pairs of male and female adults emerged from G1 were selected for G2-generation. The percentage of eggs per female, eggs hatched, larval mortality, pupal mortality, male emergence, and female emergence were observed. Each bar indicates the mean ± *SD* from the *CmHK* and *GFP* groups. Significant differences are indicated by ** (*p* < 0.05).

To investigate the transmission of the outcomes of RNAi to G2, we selected five pairs of insects from G1 and raised them separately to produce G2-generation. The study included the following parameters: eggs per female, eggs hatched, larval and pupal mortality, and sex ratio. Eggs laying and eggs hatching were significantly lower in ds*CmHK*-treated insects than those under the control treatment. The ds*CmHK*-treated insects laid 20% eggs, while the untreated insects laid 85%. Similarly, egg hatching was 48.26% in the insects treated with ds*CmHK* and 88% in the untreated ones. A considerably higher rate of larval mortality (18%) was observed under ds*CmHK* treatment in relation to the control (73%). Additionally, the male population was higher (49.33%) under ds*CmHK* than the control treatment (41%) (Figure 8).

##### G3-Generation

**Figure 9:**
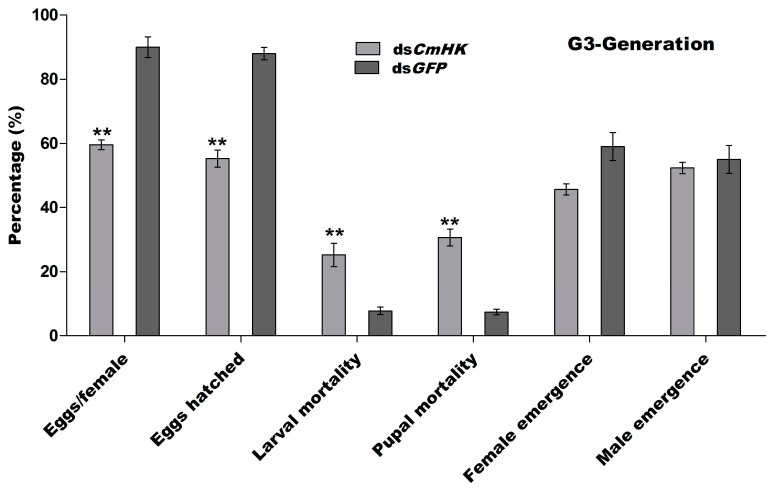
Five pairs of male and female adults were selected for G3 emerged from G2-generation. The percentage of the number of eggs per female, eggs hatched, larval mortality, pupal mortality, and adult male and female emergence were observed. Each point indicates the mean ± *SD* from *CmHK* and control groups. Significant differences are indicated by ** (*p* < 0.05).

**Figure 10:**
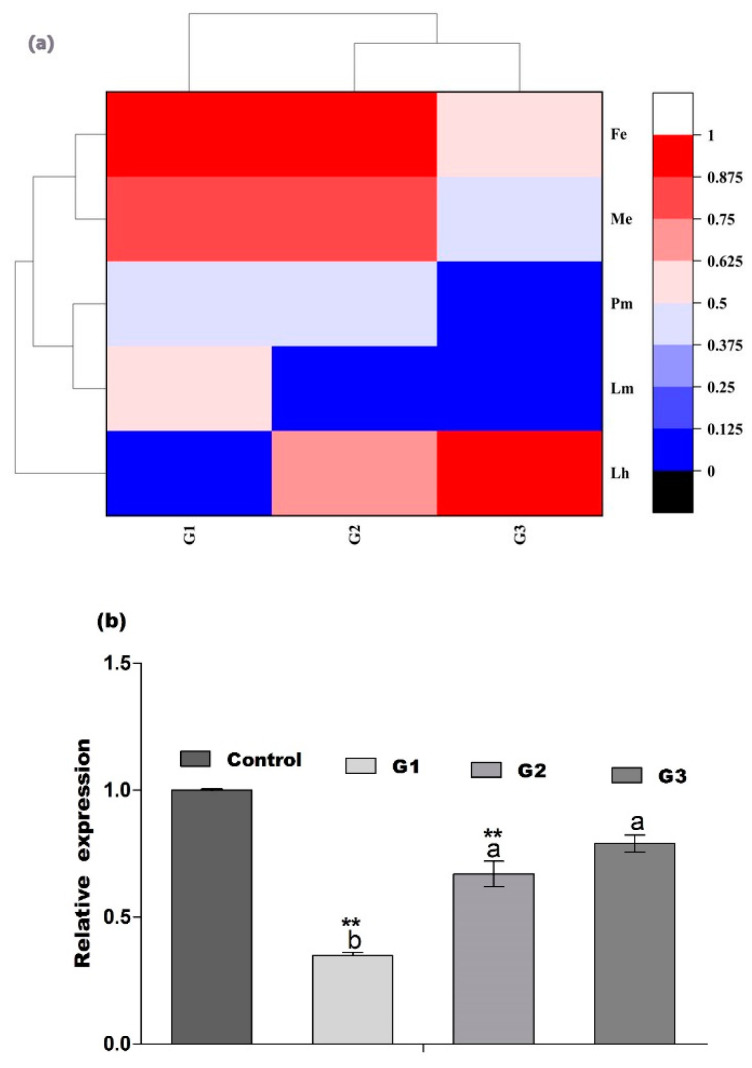
(**a**) Heat map results show the knockdown effects of the *CmHK* gene in G1, G1, and G3 generations. Lh, Larvae hatched; Lm, Larval mortality; Pm, Pupal mortality; Me, Male emergence; Fe, Female emergence. (**b**) Changes in mRNA transcript levels of *CmHK* after RNAi in G1, G2, and G3 generations. Each bar indicates the mean ± *SD*, and significant differences are indicated by ** (*p* < 0.05).

To ensure how long RNAi is effective in the ds*CmHK*-treated insect progeny, the third generation (G3) was generated by collecting five pairs of both sexes of newly emerged adults from G2 and raising them as mentioned for G1. The results indicated that the average number of eggs per female differed significantly among the treatments. However, the ds*CmHK* treatment had no effect on the eggs hatching in G3, in relation to the control. Significantly higher larval and pupal mortality was observed in those insects from the ds*CmHK* treatment as compared to the control. However, the male and female sex ratio of newly emerged offsprings did not differ significantly among treatments (Figure 9). In comparison among three generations, the heat map showed a significant reduction of eggs per female and eggs hatching, significantly increased larval and pupal mortalities, less female emergence (Figure 10a), and significantly decreased mRNA transcript levels in G1, G1, and G3 generations as compared to the control (Figure 10b).

## 4. Discussion 

The statistics obtained in this research inform discussion on dsRNA injection as it associates with pRNAi responses that may be used to control the *C. medinalis* population. Importantly, this is the first report of pRNAi effects in *C. medinalis*. This study began to investigate the characterization and knockdown of the *CmHK* gene and ended up with pRNAi effects and their transmission in three generations. The results clearly document the effects of RNAi in the ds*CmHK*-injected *C. medinalis* larvae and pRNAi response in eggs or ovaries of females that emerged from the G1, G2, and G3 generations. Sublethal effects were assessed based on the observations of significant reduction of eggs, larval and pupal mortalities, and less female emergence. Therefore, the use of the *HK* gene as an RNAi target may enable a better investigation of its silencing effects, and pRNAi response that changes mRNA transcript levels and phenotypic expression can be quantified. Based on their observations, we suggest that the *HK* gene could serve as a key model to better understand the pRNAi effects in different insects in general.

*C. medinalis* is one of the notorious pests of rice worldwide [68]. To date, *C*. *medinalis* was mainly controlled with extensive use of chemicals [57]. However, long-term use of insecticide not only leads to resistance but also affects non-target species and farmers’ health, and pollutes the environment [69]. Hai et al. identified several genes putatively involved in insecticide detoxification in *C. medinalis* larvae through transcriptome analysis [70]. Therefore, it is inevitable to find environmentally safe methods for controlling the *C*. *medinalis* population on an urgent basis. Chitin synthesis in the insect exoskeleton plays an important role in physical, biological, and chemical protection. In the present research, we identified the *hexokinase* gene from *C*. *medinalis* transcriptome database. Hexokinase plays a crucial role in glycolysis and energy metabolism through glucose singling and phosphorylation [71]. Structural domain analysis revealed that *CmHK* contains five phosphorylation sites (Figure 1). Phosphorylation plays a crucial role in protein synthesis, transportation, and enabling it to stay inbound within the cell [72]. The phylogenetic tree from CmHK and other insect hexokinases revealed that CmHK grouped with lepidopteran hexokinases, showing a high similarity with that from *O. furnacalis* (Figure 3). Moreover, three-dimensional structure predicted several C-domain and N-domain extracellular spaces and possible oligomerization (Figure 4) [73]. These results suggest that CmHK plays a crucial role in chitin formation. 

To further understand the function of *CmHK*, we investigated the expression patterns in various tissues and at different developmental stages. In insect HKs, type I was detected in almost all tissues of *B. mori*: malphighian tube and testis contained type I and type II; type I, II, and IV HKs were found in the midgut, while fat body tissues contained types I, III, and IV HKs [74]. *HK-1* was found in the head, chest, and abdominal region of *Aedes togoi* [74]. In contrast, the *HK* gene was found in the chest in *Anopheles stephensi* [75]. Our results revealed that *CmHK* was highly expressed in the cuticle, testes, as well as the ovary (Figure 2b). In relation to its expression pattern at different developmental stages, *HK-2* and *HK-3* were expressed at all developmental stages of *A. stephensi* [76], while *HK-1* was not present in the adult stages. In addition, *HK-1* was highly expressed during the last larval stages in *A. stephensi* [76]. Our results suggested that *CmHK* expression was maintained at a high level during the fourth and fifth instar larvae in *C. medinalis* (Figure 2a). In *N. lugens*, *HK* was strongly expressed in the fourth and fifth nymph stages [75], which was relatively similar to the expression pattern of *A. stephensi* [76]. Therefore, we speculate that *CmHK* also plays a role in larval to pupal transformation.

RNAi is an important and effective approach to study different gene functions. It is also used to suppress the expression of genes and to analyze gene-based biological processes [77]. For the knockdown of the target gene, it is crucial to transmit dsRNA into the insect body to silence target gene expression [78]. Mechanistic researches have stated that double-stranded ribonucleases (dsRNases), entrapment of endosome, dysfunction of core machinery, and lack of immune response contribute to reducing RNAi efficiency [79]. In lepidopteran insects, the efficiency of RNAi appeared to be lower than the coleoptera insects because dsRNA were easily degraded by RNase [80]. To investigate the RNAi efficiency, the fourth instar *C. medinalis* larvae were selected for RNAi experiments. Our results showed that phenotypic expressions, larval weight, transformation of larvae–pupae–adult emergence, oviposition, and mRNA level were disrupted after knockdown of *CmHK* (Figure 5 and Figure 6). A previous study reported that the efficiency of RNAi varied in different insect species and also maintained the gene silencing for a long time, which induced high mortality and abnormality by stunting insect growth and development [81]. Injection of dsRNA reduced oviposition in *Euschistus heros* [80]. 

Parental RNAi has been previously reported in insect pests, such as *T. castaneum* [32], *Rhodnius prolixus* [82], and in the western corn rootworm, *D. virgifera virgifera* [52]. Effects of pRNAi were also examined in both mother insects and their developing embryos by injecting dsRNA specific to the developmental gene (leg, maxillopedia, labial, and maxillary palp developing genes) [29]. To investigate the effects of the transfer of pRNAi on ds*CmHK*-treated insect progeny, a significant reduction in eggs/female, eggs hatched, female emergence, and mortality in larvae and pupae in G1, G2, and G3 generations were observed (Figure 7, Figure 8 and Figure 9 and Figure 10a). A significant down-regulation of mRNA in G1, G2, and G3 was studied (Figure 10b). Injection of dsRNA generated strong pRNAi effects in *E. heros* [80]. These findings demonstrated that with the pRNAi strategy, the down-regulation of *CmHK* by injection proved to be a useful and promising prospect for controlling the *C. medinalis* population.

## 5. Conclusions

Taken together, *CmHK* was identified and characterized based on the transcriptome database of *C. medinalis*. The RT-qPCR analysis suggested that *CmHK* was highly expressed in the cuticle, testes, and ovary. The expression pattern of *CmHK* at developmental stages showed that it was predominantly expressed in the fourth and fifth instar larvae. The silencing of *CmHK* significantly inhibited weight gain and normal growth of the ds*CmHK*-injected larvae, pupation, and reduced population of females. Furthermore, the pRNAi of *CmHK* experiments showed a significant reduction in egg laying, eggs hatched, mortality of both larvae and pupae, and female emergence in three generations. However, further studies are suggested to investigate the lethal effects of the *HK* gene on *C. medinalis* and its generations by dsRNA feeding bioassays. 

## Figures and Tables

**Table 1:** Primer information for cloning and expression analysis of *CmHK*.

Primer Name	Primer Sequence	Primer Usage
HK-F	TCGCAGAAGAGGTATTGACTCA	RT-PCR
HK-R	GATATGACTCGACGTTGGTGTT	
HK-iF	AGGTCCTGCATATGACAGACAAAC	dsRNA Synthesis
HK-iR	CACAATGTGTATGAGGAACGTCT	
HK-dsF	taatacgactcactatagggAGGTCCTGCATATGACAGACAAAC	
HK-dsR	taatacgactcactatagggAGACGTTCCTCATACACATTGTG	
GFP-iF	GCCAACACTTGTCACTACTT	
GFP-iR	GGAGTATTTTGTTGATAATGGTCTG	
GFP-dsF	taatacgactcactatagggGCCAACACTTGTCACTACTT	
GFP-dsR	taatacgactcactatagggGGAGTATTTTGTTGATAATGGTCTG	
HK-qF	ACTCACACGCTACATCTATCG	RT-qPCR
HK-qR	GACGCCAGTACCAGTCATAA	
Actin-F	ATGGTCGGCATGGGACAG	
Actin-R	GAGTTCATTGTAGAAGGTGT	

Note: the lowercase letters in the primers represent the T7 promoter sequence.
